# Comparison of Postural Sway, Plantar Cutaneous Sensation According to Saccadic Eye Movement Frequency in Young Adults

**DOI:** 10.3390/ijerph17197067

**Published:** 2020-09-27

**Authors:** Youngsook Bae

**Affiliations:** Department of Physical Therapy, College of Health Science, Gachon University, 191 Hambangmoe-ro, Yeonsu-gu, Incheon 21936, Korea; baeys@gachon.ac.kr; Tel.: +82-32-820-4324

**Keywords:** center of pressure, plantar cutaneous sensation, postural sway, saccadic eye movement, healthy adults

## Abstract

The crossover trial study aimed to identify the saccadic eye movement (SEM) frequency to improve postural sway (PS) and plantar cutaneous sensation (PUS) in young adults. The 17 participants randomly performed 0.5-, 2-, and 3-Hz SEM. The SEM frequency was determined to allow the target to appear once per 2 s (0.5 Hz), twice per second (2 Hz), or thrice per second (3 Hz). SEM performance time was 3 min with a washout period of 5 min. PS and PUS were measured at baseline and during 0.5-Hz, 2-Hz, and 3-Hz SEMs using a Zebris FDM 1.5 force plate. PS was determined by measuring the sway area, path length, and speed of center of pressure (COP) displacement, and PUS was determined via the plantar surface area (PSA). In PS parameters, there was a significant difference among the SEM frequencies in the COP_sway area_ PSA_left foot_ and PSA_right foot_. Compared to that at baseline, COP_sway area_ decreased at 0.5 Hz and 2 Hz, while PSA_left foot_ and PSA_right foot_ increased at 2 Hz. These results suggest that 2 Hz SEM may improve PS and PSA.

## 1. Introduction

Balance is affected by various activities and interventions, and improving balance is an important concern [1]. In particular, interventions are needed to improve balance in order to prevent disability in unimpaired populations [2,3]. Lack of visual information in the environment requires re-weighting of other sensory systems, such as the vestibule and proprioception, to control posture [4]. In addition, previous studies have demonstrated the influence of eye movement on postural control during upright standing [5,6,7,8]. As such, visual information plays an important role in postural control and the maintenance of postural balance.

Visually tracking a target while moving quickly from one target to another reduces the postural sway (PS) [7]. In addition, voluntary saccadic eye movement (SEM) is a quick, simultaneous movement of both eyes between two or more phases of fixation in the same direction [9] and requires visual attention [10]. The continuous extraocular muscle afferent caused by external stimulation is also related to the postural balance [11]. Therefore, to maintain stable upright postural control, attention and external sensory stimulation as well as postural control systems are required. In particular, PS is decreased during SEM rather than during a fixed gaze in young adults [7,12], older adults [8,13], and people with multiple sclerosis [14]. Such findings indicate that SEM relative to the moving target is used to determine the body position in space [11], and that it plays an important role in controlling PS during upright standing.

PS in a quiet, upright standing position is quantified by measuring the time course of the center of pressure (COP). COP represents the point of application of the ground reaction force vector. COP displacement is of critical importance for understanding the musculoskeletal and neural system of postural control [15,16]. There is an association between balance improvement and the facilitation of sensory feedback related to the activation of plantar cutaneous mechanoreceptors [17]. Plantar cutaneous mechanoreceptors are extremely important in the maintenance of postural control, due to their direct interaction with the support surface [18]. Plantar sensation is important for maintaining a normal standing balance. Therefore, PS is related to plantar sensation [19]. Moreover, previous studies have shown decreased PS and increased plantar surface area (PSA) of the foot in contact with the ground at a SEM frequency of 0.5 Hz in young adults and older persons [8,12,13]. Deterioration of plantar cutaneous sensation information of the foot support on the ground worsens postural balance [19]. The area of COP excursions may reduce in an effort to curtail exploratory postural behavior given the altered afferent input from plantar receptors [20]. Therefore, SEM stimulation may be a useful method for decreasing PS and increasing PSA for older adults and neurologic patients with poor balance.

The decrease in PS during SEM is believed to provide not only visual attention but also external sensory stimulation. Previous studies reported that SEM is related to visual attention [10,21]. However, studies on the application of SEM stimulation to improve postural balance are lacking. In addition, the effect of applying the frequency of SEM stimulation to improve postural balance is unclear. Therefore, the aim of the present study was to identify the SEM frequencies that reduce PS and increase PSA in young adults. Considering the findings of previous studies, this study was designed to determine which SEM frequencies (0.5, 2, or 3 Hz) are effective in improving postural balance.

## 2. Materials and Methods

### 2.1. Research Design

This pilot study was designed as a crossover trial. Participants performed SEM randomly at 0.5 Hz, 2 Hz, and 3 Hz depending on the code shown in a sealed, opaque envelope. A Latin square design was used to eliminate the order effect caused by repeated experiments. Randomization was performed by an investigator who was not related to the recruited participants. All procedures were approved by Gachon University’s Institutional Review Board (clinical trial registration number: KCT 0004386) were conducted in accordance with the 1975 Helsinki Declaration. The data for the study were collected from October to November 2019.

### 2.2. Participants and Procedure

Seventeen young adults (mean age, 23.06 years) participated in this study. Participants were recruited from the University of Gachon student community via advertisements on posters and websites and selected according to the inclusion and exclusion criteria. Inclusion criteria were adults between the age of 20–30 with no history of falls or dizziness, no surgical history or no pain and discomfort in the lower extremities, performed over 180 min of moderate or mild physical activity per weeks for more than 4 weeks, and able to perform the intervention for more than 30 min. Individuals who had previous balance and neurologic or vestibular impairment were excluded. Before the experimental process, the participants were informed in detail about the study procedure and safety, and they signed written informed consent.

At the beginning of this study, 18 young adults were screened. However, one did not meet the study’s inclusion criteria. The random allocation was performed by a researcher who was not involved in the recruitment process. The demographic characteristics of the participants were examined, and baseline measurements were performed before random allocation. All participants were trained to familiarize themselves with the method of performing SEMs prior to the study. SEMs were performed for 3 min, which corresponds to the amount of time required to prevent dizziness or discomfort for the participants [8], and 5 min of a washout was provided between interventions. Participants were encouraged to walk freely during the washout period.

All data collection took place at a university laboratory. Although researchers were aware of the allocated groups, outcome assessors and data analysts were blinded to the allocation. This study used G*Power 3.1.7 software to calculate the sample size, which was determined on the basis of a power of 80%, a significance level at *p* < 0.05, we calculated that a sample size of 16 participants was necessary [22].

### 2.3. Intervention (Saccadic Eye Movement)

**Figure 1:**
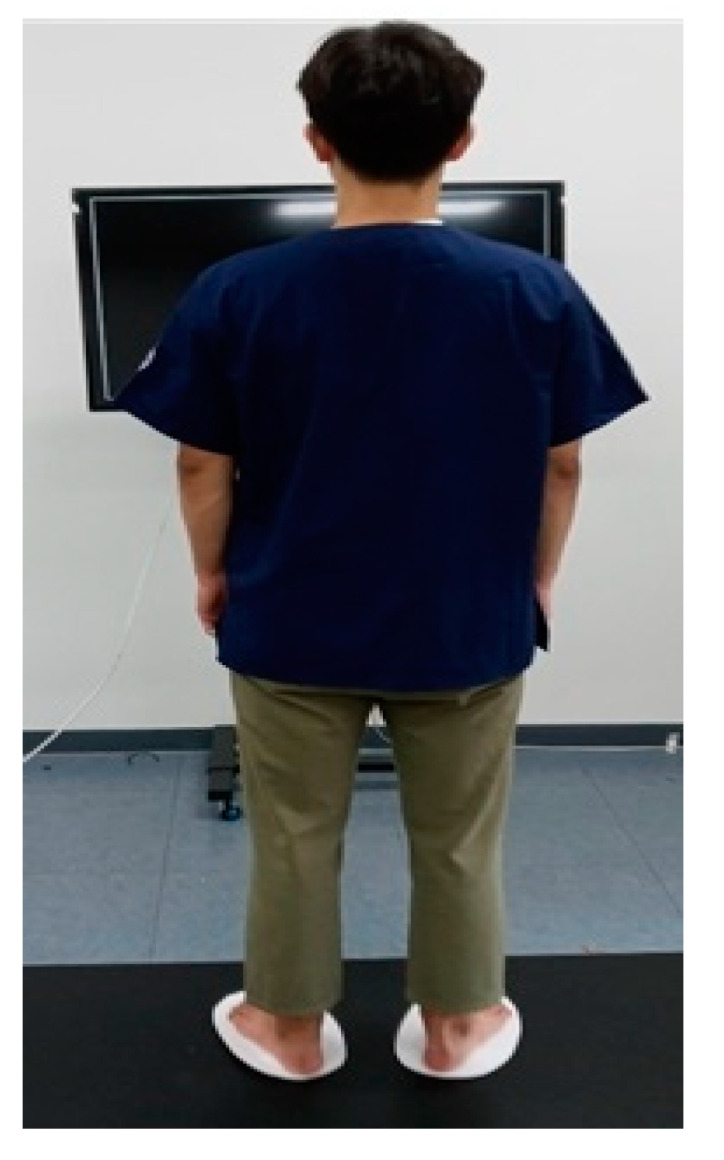
Intervention of saccadic eye movement.

All participants underwent SEM with the blinded assist researcher each trial. All participants stood barefoot on the force plate in the same position. Each participant stood on both feet in a neutral position with eyes open. The arms were placed in a relaxed position parallel to the trunk. The participants then performed SEMs by only moving their eyeballs, without moving the trunk or head, following the movement of the target appearing on the monitor screen 1 m in front of them. The target was a red dot with a 2 cm diameter on a black background. The target was generated by flash software and displayed on an LCD monitor (97.9 cm × 58.3 cm, LG, South Korea) screen (Figure 1).

To measure the baseline, the target was fixed for approximately two minutes in the middle of the monitor screen to focus the eyes of the participants in one place. To measure the SEM, the target appeared in one place on the screen, disappeared, and immediately reappeared in another position, which caused the participants’ eyes to saccade the target. Targets were set to appear randomly in horizontal and vertical directions. Fixation of the head of the participants during the intervention ensured that the total visual distance on both the left and right sides was set to be approximately 11° [8,12].

Aguiar et al. [13] found that there was no difference in the reduction of PS amplitude when SEMs performed at 0.5 and 1.1 Hz in young adults. This study used frequencies of 0.5, 2, and 3 Hz, because participants complained of dizziness above 4 Hz in a preliminary test of three individuals before conducting the study. The targets appeared once every 2 s, twice per second, or three times per second for the 0.5, 2, and 3 Hz conditions, respectively.

### 2.4. Outcome Measures

The participants’ general characteristics were measured pre-assessment. PS and PSA were measured during baseline and at 0.5-, 2-, and 3-Hz SEM simultaneously.

#### 2.4.1. Postural Sway

**Figure 2:**
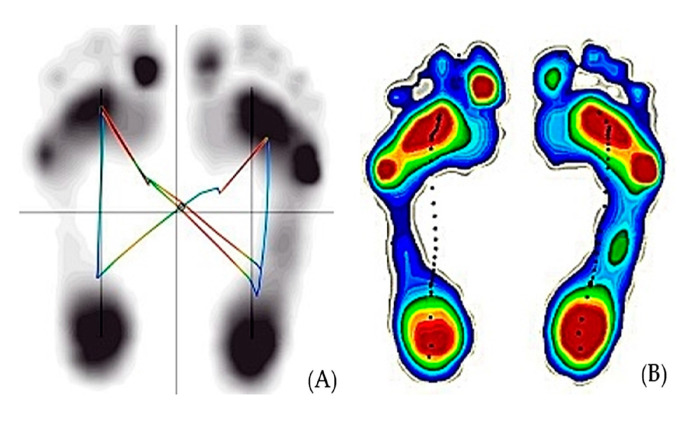
The data presented by the Zebris force plate. (**A**) Center of gravity (COG) parameters; (**B**) plantar surface area.

PS was measured as a COP displacement. COP parameters are reliable in terms of intra-subject repeatability and can detect significant individual subject movement patterns [23]. COP displacement was measured with a valid and reliable force platform (Zebris FDM 1.5, Zebris Medical GmbH; Isny im Allgäu, Germany) [24]. The Zebris force plate was used for data collection and analysis when measurements were taken in a standing position. The platform size (length × width × height) was 1580 mm × 605 mm × 21 mm. It incorporates 11,264 sensors on an area of 64 × 176 cm, giving a resolution of approximately 4 sensors per square centimeter and a sampling rate of 100 Hz. The software was provided with the equipment calculated COP parameters from the raw data delivered by the platform. In this study, COP parameters, including the sway area (mm^2^), path length (cm), and speed (cm/s) of COP displacement, were measured as primary outcomes (Figure 2A). COP parameters were reliable for 30 s in a bipedal position [25,26]. Therefore, the sampling duration of COP measurements was set to 60 s, as suggested by Carpenter et al. [27], and was measured from 60 s after performing the SEM.

#### 2.4.2. Plantar Cutaneous Sensation

To confirm changes in plantar cutaneous sensation, the PSAs of both feet were measured as the area in contact with the floor in the pedobarographic image displayed on the monitor screen during the SEM intervention. The software generated the data on PSAs beneath both feet from the raw data delivered by the platform. PSAs measured the contact area of the left and right feet (mm^2^) (Figure 2B). PAS measurements were taken for 60 s during SEM.

### 2.5. Statistical Analysis

All statistical analyses were performed using SPSS 25 (IBM Corp., Armonk, NY, USA), and descriptive statistics were used for the general demographics of the participants. The dependent variables between the groups were compared using a one-way repeated ANOVA. The baseline and SEM frequency were compared by paired t-test. The effect sizes of the interaction frequency were calculated as Eta-squared (η2) to determine meaningful changes between groups: an effect size of up to 0.02 indicated a small change; 0.13, a moderate change; and 0.26, a large change [28]. Additionally, the Bonferroni correction method was used to correct errors that may have occurred in comparisons between SEM trials. The new significance level was 0.05/(comparison number) based on Bonferroni correction [29]. Therefore, in this study, the adjusted significance level was 0.016. All variables are expressed as means ± SD.

## 3. Results

The average age of the 17 participants was 23.35 years (range 20–28 years), and the general demographics are summarized in Table 1.

For PS parameters, the greatest decrease in COP_sway area_ was at 2 Hz, and there was also a significant difference between SEM frequency groups (*p* = 0.002, η^2^ = 0.344). The PSAs of the left foot (*p* = 0.002, η^2^ = 0.316) and right foot (*p* = 0.002, η^2^ = 0.324) were significantly increased at 2 Hz (Table 2).

In the post-hoc comparison, compared to that at baseline, COP_sway area_ was decreased at 0.5 Hz (*p* = 0.002) and 2 Hz (*p* < 0.001), while the PSAs of the left foot (*p* < 0.001) and right foot (*p* = 0.001) were increased at 2 Hz (Table 3). These results indicate that SEMs at 0.5 and 2 Hz SEM can affect PS, and 2Hz SEM, in particular, has a more positive effect on enhancing plantar cutaneous sensation information.

## 4. Discussion

In this study, there was a significant difference in COP_sway area_ at 0.5-Hz SEM. This proves the reliability of this study with results similar to those of previous studies. Moreover, at 2 Hz, COP _sway area_ was significantly decreased and PSAs in both feet were significantly increased. These results prove that 2-Hz SEM significantly decreased PS and improved plantar cutaneous sensation information. Previous studies have confirmed vision as an important part of postural control [30,31]. To maintain balance, it constantly reconsidered input from other parts of the balance system and responses from the motor cortex. This means postural control, and is an ability to maintain the line of gravity of a body within the base of support with minimal postural sway wherein the body moves constantly [32]. In this study, PS decreased at 0.5, 2, and 3 Hz. This result demonstrated that SEM stimulation had a positive effect on postural balance, similar to previous studies [8,12,13].

Visual signals improve balance, and optic flow and eye movements provide information about body position relative to a target [33,34,35]. Ocular movements may modify postural control in the maintenance of upright standing posture in humans [36]. In this study, COP_sway area_ significantly decreased from a baseline value of 80.24 mm^2^ to 41.23 mm^2^ at 0.5 Hz and 21.58 mm^2^ at 2 Hz. This result shows that 0.5- and 2-Hz SEM provide sensory stimulation to induce postural control. The decrease in the COP_sway area_ may suggest that postural control increased from SEM stimulation. Our finding further confirmed that SEM-induced eye movement in a static standing position positively influences PS and corroborated the results of previous studies showing a decrease in PS at an SEM frequency of 0.5 Hz in healthy adults [19,20]. A previous study suggested that visuospatial attention is an important mechanism in generating voluntary SEMs [15]. Precise visual tasks may require the brain to control the synergistic relationship between eye and body movements instead of individual eye and body movements [37]. Applying sufficient mechanical vibration to activate the large afferent fibers of the extraocular muscle or tendon causes the extraocular signal to form extraocular perception, which is an important factor for postural control [11,38]. In the oculomotor system, it provides the only extra-retinal signal about eye position that is available without delay, and it is shown to be the most important extra-retinal source of information for motor activity [11,39]. In a previous study, body sway was more reduced during SEM than gaze fixation [13]. This suggests that extraocular input to eye positioning is an important signal that should be integrated with perceptual optic nerve flow processing to control body sway [35]. In this study, COP_sway area_ significantly decreased at 2 Hz compared to 0.5 Hz. There were significant differences between groups, and the effect size was also large (0.344). Therefore, 2 Hz may be more efficient than 0.5 Hz as a SEM frequency to stimulate extraocular muscles and improve visual attention while performing precise visual tasks.

A decrease in cutaneous information from the feet has shown to increase PS [40]. PS refers to the ability to stand for as long as possible on a force plate in situations where the somatosensory conditions change occasionally [41]. Significant interactions were found between sensation and vision for the double-limb COP area. The COP_sway area_ may have reduced in an effort to curtail exploratory postural behavior given the altered afferent input from plantar receptors [20]. Furthermore, plantar sensation can be identified as an important factor in maintaining balance against external stimuli in an upright standing position, and the wider the plantar surface of the foot in contact with the ground, the greater the amount of cutaneous sensation information input that can be received. Attention is required sensory integration for standing postural control in the elderly and young people [42,43]. In this study, the PSAs of both feet were significantly wider during 2-Hz SEM compared to baseline. Additionally, PSA was significantly increased at 2 Hz compared to 0.5 and 3 Hz. The effect sizes for the left and right feet were large: 0.264 and 0.344, respectively. Therefore, the increase in attention due to 2-Hz SEM may have increased the integration of visual stimuli and plantar proprioception due to the increase in PSA and the decrease in PS during SEM application. Moreover, based on these results, 2-Hz SEM may be an effective frequency for inducing attention and improving balance.

This study has several limitations. First, this study suggests that even if the subject has sufficiently practiced SEM, further studies need to confirm that eye tracking is performed correctly during SEM for the effect of SEM on postural balance. Second, the washout period (5 min) was short to eliminate the sequencing effect caused by repeated experiments. Finally, PS was measured only during the SEM while maintaining a standing position. Therefore, it is necessary to confirm these changes in PS and lower extremity muscle activity according to SEM performance on unstable surfaces that require greater balancing abilities.

Despite these limitations, this study has several advantages. This is the first study to identify the most efficient SEM frequencies for improving balance. Therefore, it provides the basis for further research on SEM to promote or improve balance ability. Moreover, this study has clinical significance because the balance ability was verified through SEM frequency.

## 5. Conclusions

Our findings show that COP_sway area_ was reduced in 0.5 and 2 Hz saccadic eye movement compared to baseline (gaze fixation). Plantar sensation of both feet increased in 2 Hz SEM. This result indicates that 2 Hz SEM is an effective and feasible intervention for improving postural balance, and it is proposed as an intervention for promoting postural stability.

## Figures and Tables

**Table 1:** Baseline characteristics of the participants.

	Total (*n* = 17)	Male (*n* = 6)	Female (*n* = 11)
Age (years)	23.35 ± 2.58	22.50 ± 2.43	23.82 ± 2.68
Height (cm)	167.18 ± 8.49	175.17 ± 2.48	162.82 ± 7.29
Weight (kg)	63.41 ± 13.60	68.50 ± 8.96	60.64 ± 15.23

**Table 2:** Comparisons of postural sway and plantar surface area between baseline and 0.5, 2, and 3 Hz saccadic eye movement.

	Baseline	0.5 Hz	2 Hz	3 Hz	Between Frequency			
					F	*p*	η^2^	Adj Sig ^†^
Postural sway parameter								
COP_sway area_ (mm^2^)	80.24 ± 57.99	41.23 ± 25.00	21.58 ± 16.03	44.82 ± 43.21	8.407	**0.002**	0.344	**Yes**
COP_path length_ (cm)	18.41 ± 5.46	15.42 ± 3.36	14.865 ± 2.35	15.17 ± 3.31	4.551	0.028	0.221	No
COP_speed_ (m/s)	0.62 ± 0.19	0.51 ± 0.10	0.49 ± 0.07	0.51 ± 0.10	5.123	0.022	0.243	No
Plantar surface area								
Left foot (mm^2^)	99.88 ± 15.54	102.88 ± 30.75	119.00 ± 16.79	112.41 ± 14.03	5.739	**0.011**	0.264	**Yes**
right foot (mm^2^)	100.59 ± 13.15	109.00 ± 13.99	115.88 ± 16.21	112.35 ± 9.32	7.694	**0.002**	0.325	**Yes**

COP: center of pressure, PSA; plantar surface area, ^†^ adjusted significance (significance alteration *p* value to 0.016) are shown bold.

**Table 3:** Post hot test for intra-saccadic eye movement (SEM) frequency pair-wise comparison.

	Baseline vs. 0.5 Hz		Baseline vs. 2 Hz		Baseline vs. 3 Hz	
	MD	*p*	MD	*p*	MD	*p*
Postural sway parameter						
COP_sway area_ (mm^2^)	39.00	**0.002**	58.64	**0.000**	35.41	0.048
COP_path length_ (cm)	2.98	0.039	3.55	0.030	3.24	0.034
COP_speed_ (m/s)	0.11	0.032	0.12	0.019	0.10	0.047
Plantar surface area						
Left foot (mm^2^)	3.00	0.684	19.11	**0.000**	12.52	0.017
right foot (mm^2^)	8.41	0.053	15.29	**0.003**	10.29	0.018

MD: Mean difference; Statistical significance (adjusted *p*-value to 0.16) compared to baseline are shown bold.
